# Pathogenic *Leptospira* and their animal reservoirs: testing host specificity through experimental infection

**DOI:** 10.1038/s41598-020-64172-4

**Published:** 2020-04-29

**Authors:** Colette Cordonin, Magali Turpin, Matthieu Bringart, Jean-Loup Bascands, Olivier Flores, Koussay Dellagi, Patrick Mavingui, Marjolaine Roche, Pablo Tortosa

**Affiliations:** 1 Unité Mixte de Recherche Processus Infectieux en Milieu Insulaire Tropical (UMR PIMIT), Université de La Réunion, CNRS 9192, INSERM 1187, IRD 249. Plateforme de recherche CYROI, Sainte-Clotilde, La Réunion France; 2 Unité Mixte de Recherche Diabète Athérothrombose Thérapies Réunion Océan Indien (UMR DéTROI), INSERM U1188, Université de La Réunion. Plateforme de recherche CYROI, Sainte-Clotilde, La Réunion France; 3 Unité Mixte de Recherche Peuplements Végétaux et Bioagresseurs en Milieu Tropical (UMR PVBMT), Université de la Réunion, Saint-Pierre, La Réunion France; 4 Present Address: Department of International Affairs, Institut Pasteur, Paris, France

**Keywords:** Evolutionary biology, Microbiology

## Abstract

Leptospirosis is caused by pathogenic *Leptospira* transmitted through contact with contaminated environments. Most mammalian species are infectable by *Leptospira* but only few act as efficient reservoir being capable of establishing long term kidney colonization and shedding *Leptospira* in urine. In Madagascar, a large diversity of pathogenic *Leptospira* display a tight specificity towards their endemic volant or terrestrial mammalian hosts. The basis of this specificity is unknown: it may indicate some genetically determined compatibility between host cells and bacteria or only reflect ecological constraints preventing contacts between specific hosts. In this study, *Rattus norvegicus* was experimentally infected with either *Leptospira interrogans, Leptospira borgpetersenii* or *Leptospira mayottensis* isolated from rats, bats or tenrecs, respectively. *Leptospira borgpetersenii* and *L. mayottensis* do not support renal colonization as featured by no shedding of live bacteria in urine and low level and sporadic detection of *Leptospira* DNA in kidneys. In contrast 2 out of the 7* R. norvegicus* challenged with *L. interrogans* developed renal colonization and intense *Leptospira* shedding in urine throughout the 3 months of experimental infection. These data suggest that host-*Leptospira* specificity in this biodiversity hotspot is driven at least in part by genetic determinants likely resulting from long-term co-diversification processes.

## Introduction

Leptospirosis is a bacterial zoonosis of global importance that is transmitted either by direct contact with infected animals or most commonly by indirect contact through an environment contaminated with the urine shed by infected mammalian reservoirs. Pathogenic *Leptospira* are highly diversified spirochetes currently composed of ten identified species, namely *Leptospira alexanderi*, *Leptospira alstoni*, *Leptospira borgpetersenii*, *Leptospira interrogans*, *Leptospira kirschneri*, *Leptospira kmetyi*, *Leptospira mayottensis*, *Leptospira noguchii*, *Leptospira santarosai*, and *Leptospira weilii*^1,2^. However, the *Leptospira* diversity is likely overlooked^3^ and full genome sequencing of environmental samples will surely increase the number of recognized pathogenic species in the next future.

Rodents are considered as important reservoirs of *Leptospira*, and have hence been investigated in a number of environmental settings^4–7^. Molecular typing of *Leptospira* hosted by rats from Reunion Island and Seychelles have revealed the dominance of a single species, *L. interrogans*, represented by three Sequence Types (STs), two of them being reported in different locations worldwide^4,5^. The very limited diversity of these rat-borne leptospires together with cosmopolitan lineage distribution support a recent introduction of *L. interrogans* in these oceanic islands, possibly concomitant with rat introduction.

Besides rodents, a wide range of mammals can actually host *Leptospira* spp. (*i.e*. support kidney colonization), as confirmed by a thorough investigation of wild mammals from southwestern Indian Ocean (SWIO) islands^8^. Volant and terrestrial small mammals have been investigated in Madagascar and surrounding islands where bacterial isolation and molecular investigations have uncovered a remarkable diversity of mostly original *Leptospira* lineages^2,9–15^. Contrary to studies that indicate the lack of host specificity and a possible geographic structuring among *Leptospira* spp.^16,17^, detailed studies conducted in Madagascar highlighted that Malagasy *Leptospira* show a marked specificity towards their hosts, as best exemplified by the exploration of tenrec and bat reservoirs^12,13^. Indeed, each bat considered at the family or genus level in Madagascar shed *Leptospira* belonging to lineages that have co-diversified with their hosts, lineages which can hence be considered as endemic to Madagascar^13^. Similarly, *L. mayottensis*, a leptospiral species of public health concern on Mayotte island^2,10,15^, is found only in Tenrecidae in Mayotte and Madagascar^12,15^, suggesting a strong affinity of *L. mayottensis* for this mammalian family. Host specificity patterns have also been reported in other African environmental setups. In Benin, *L. kirschneri* is associated with shrews while *L. interrogans* may be found in several host species although mainly in *Rattus norvegicus*^18^. In Tanzania, unique lineages of *L. borgpetersenii*, different from references sequences, are found in cattle, while *L. kirschneri* can be found in several ruminant species^9^.

Noteworthily, rats in Mayotte, an introduced species, are infected with a handful of *Leptospira* species, suggesting that rats can be a reservoir of a wider diversity of *Leptospira* than endemic mammals^13^. However, the ability of rats to harbor endemic *Leptospira* lineages is not so clear. Thus, in 2016, a study conducted in Mayotte did not report *L. mayottensis* renal colonization in any of the 289 screened rats. However in Madagascar, this species was identified in a small percentage of infected *Rattus* through the development of a sophisticated screening strategy allowing the investigation of multiple infections^19^. Of note, *L. mayottensis* was found in *Rattus* as co-infections only. Authors claimed that rats may have acquired *L. mayottensis* infection through direct or indirect contact with tenrecs. These observations question the role of rats in the transmission of endemic strains of *Leptospira* in humans on SWIO islands and the ability of strains, likely host-specific, to be sheltered by introduced reservoirs such as rats. Beside these observations, endemic *Leptospira* seem to exhibit a strong specificity towards their hosts that may result from ecological drivers such as the absence of contact between endemic and introduced mammal hosts impeding trans-contamination of rats by endemic *Leptospira*. Alternatively, co-evolutionary processes taking place during the extraordinary radiation of tenrecs, rodents and bats in Madagascar during the last 20–30My^20,21^ may have led to specific host-parasite interactions driving strong host specificity^13^.

*Rattus norvegicus* is a well-known reservoir of *Leptospira*, including in SWIO, and has been proven as a good experimental model to study chronic infection^22–24^. To bring a first insight on the mechanisms responsible for the host specificity observed on the field, we tested whether isolates of *L. borgpetersenii* and *L. mayottensis*, obtained from bat and tenrec specimen, respectively, and considered endemic to SWIO, are able to replicate in a genetically different host such as *R. norvegicus*. An isolate of *L. interrogans* harboring a worldwide distributed ST and obtained from a rat from Reunion Island was used as a positive control.

## Materials and methods

### Animal experiments and ethical statement

Seven- to eight-week-old female Wistar rats (Janvier Labs, Le Genest, France) were housed by 4 in enriched cages at the CYROI platform’s animal facility. The appropriate number of animals was calculated using the Resource Equation method^25^. Experiments were conducted following the guidelines of the Office Laboratory of Animal Care at the CYROI platform. All animal procedures were performed in accordance with the European Union legislation for the protection of animals used for scientific purposes (Directive 2010/63/EU) and approved by the French Ministry of Sciences and Superior Education under the number APAFIS#8773–2016111615105111 v2.

### Bacterial isolates

*Leptospira* were isolated from kidneys of field-trapped volant and terrestrial small mammals. *Leptospira interrogans* was isolated from *Rattus rattus* on Reunion Island (2013RR GLM983)^5^, *L. mayottensis* was isolated from *Tenrec ecaudatus* on Mayotte (2014TE MDI222)^15^, and *L. borgpetersenii* was isolated from an insectivorous bat, *Triaenops menamena*, endemic to Madagascar (2014TM FMNH228863)^26^. All isolates were genotyped through Multi Locus Sequence Typing (scheme 3). Of note, the used *L. interrogans* isolate has been previously genotyped as Sequence Type 02 (ST02, MLST scheme#3), an ST that is widespread in both *R. rattus* and *R. norvegicus* on Indian ocean islands such as Seychelles and Reunion^4,5^. The *L. interrogans* and *L. mayottensis* isolates were selected for being associated with acute leptospirosis human cases on Reunion Island and Mayotte, respectively. The *L. borgpetersenii* isolate exhibited an original ST that has never been identified in any human leptospirosis case. Although characterized as *L. borgpetersenii*, it is important to mention that *Leptospira* shed by Malagasy bats have very unique genetic profiles and display strong host-specificity: these lineages form monophyletic clades composed of lineages shed by bats belonging to the same genus^13^. All *Leptospira* isolates were grown in Ellinghausen-McCullough-Johnson-Harris (EMJH) liquid medium (Difco, Detroit, MI, USA) supplemented with albumin fatty acid supplement (AFAS) (purchased at AO/OIE and National Collaborating Centre for Reference and Research on Leptospirosis Academic Medical Center, Department of Medical Microbiology, Amsterdam) and 5-fluorouracil (5-Fu) at 28 °C (for detailed protocol, see http://dx.doi.org/10.17504/protocols.io.ifccbiw). The isolates were stored at −80 °C or in liquid nitrogen for three to four years and their virulence was restored by two serial passages in hamsters, then stored at −80 °C and passaged less than 5 times before the start of the experiments^27^. The virulence of the three isolates has been tested on hamsters in a previous study in our lab and revealed a higher virulence of *L. interrogans* compared to the two other strains^27^.

### Infection and sample collection

Challenged rats were inoculated intraperitoneally (i.p.) with 1.5 × 10^8^ bacteria of *L. interrogans* (n = 7 animals), *L. mayottensis* (n = 8) or *L. borgpetersenii* (n = 7) resuspended in 500 µl sterile phosphate buffered saline (PBS) 1×. This inoculated bacterial load was chosen following a pilot study because it induced live *Leptospira interrogans* excretion in the urine of rats after four weeks post-infection. Although i.p. inoculation is not a natural route of infection, this method is acknowledged as the most convenient method to inject a reproducible volume of inoculum^28^. Control rats (n = 8) were injected i.p. with 500 µl sterile PBS 1X. Animals were monitored for three months and urine samples were collected once a week during the whole experiment. After three months, animals were anesthetized with 50 mg/kg pentobarbital injected i.p. and subsequently euthanized by cardiac puncture. The animals were then transcardially perfused with PBS 1X to flush out the blood. The kidneys were then collected and processed for further experiments or immediately frozen at −80 °C.

### Bacterial load measurement

A piece of up to 25 mg of frozen kidney was lyzed in ATL buffer (Qiagen, Germany) for 3 hours minimum. DNA from frozen kidneys and from urine samples (30 to 100 µl) was extracted using DNeasy Blood & Tissue Kit (Qiagen, Germany). The DNA of pathogenic *Leptospira* was quantified in each sample by a probe-specific real-time PCR using Quantinova probe PCR mix (Qiagen, Germany). All samples were triplicated and samples with at least two replicates leading to a PCR amplification at Ct <45 were considered positive. Results are expressed as genome copies per milligram of tissue for kidney samples or as genome copies per microliter for urine samples. The genome size of *L. interrogans* strain Fiocruz L1–130 was used to calculate the number of genome copies^29^.

### Viability of excreted leptospires

2 ml of EMJH liquid medium supplemented with AFAS and 5-Fu were inoculated with 30 to 50 µl urine. Cultures were observed regularly for three months to check the presence of cultivable leptospires under a dark-field microscope (Axio Lab.A1, Zeiss France).

### Histological studies

For each animal, a whole kidney was fixed in 4% paraformaldehyde for 24 to 48 hours. Kidneys were then serially dehydrated using a spin tissue processor STP120 (Myr, Spain) as follows: three baths of Ottix Shaper at room temperature (10 minutes, 1 hour and 1.5 hours) were followed by 6 baths of Ottix Plus at room temperature (1 hour, 1.5 hour twice, 2 hours and 2.5 hours), and 2 paraffin baths at 62 °C (1.5 hour and 2.5 hours). Kidney tissues were then embedded in paraffin. Four- to six-µm-thick paraffin kidney sections were deparaffinized in three successive xylene baths, 10 minutes each, rehydrated in 3 successive ethanol baths (100%, 70% and 50%, 2 minutes each) and in distilled water bath, 2 minutes each. Rehydrated sections were eventually stained with hematoxylin and eosin, and observed using a digital slide scanner (NanoZoomer S60, Hamamatsu, France). Altogether, a minimum of three distinct animals were examined for each *Leptospira* infection and control.

### Analysis of *Leptospira* DNA detection in urine

Regarding DNA detection in urine, two approaches were implemented to cope with particularities observed in the sampled dataset: a high number of urine samples without any detected copy of *Lesptospira* genome, leading to zero-inflation in the distribution of the number of detected copies (Y = 0, where Y is the number of copies detected in one urine sample), and strong variability across tested rats, indicative of inter-individual effects. Hence, we first analyzed the overall load measured over the study period, that is the total number of genome copies (Y_tot_) for differences in variance and mean across groups. Means were compared in a simple ANOVA after log-transformation in the latter case to improve the normality of the distribution (log(Y_tot_ + 1)). Residuals were checked for normality and variance homogeneity. Second, we built a linear mixed model to account for the different experimental variables that are strain and rat identity as well as time after infection (WPI: week post-infection), while taking advantage of the full variability of the sampled dataset. A Gaussian model of the transformed unitary response (log(Y + 1)) was constructed while accounting for zero-inflation in the distribution. *Leptospira* strain identity and WPI were used as fixed-effects variables while rat identity nested within strain was used as a random-effect variable in both the conditional and the zero-inflated components of the model. The final model was defined and validated after model comparison with varying structure of the fixed and random effects and for residual normality and homogeneity.

## Results

### Experimentally infected rats show no clinical signs

**Figure 1:**
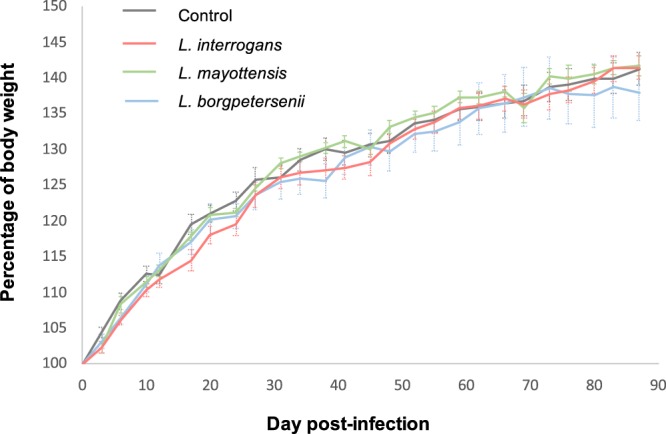
Rats monitoring. Growth curves of control rats (black) and rats infected with either *L. interrogans* (red), *L. mayottensis* (green) or *L. borgpetersenii* (blue). Results are expressed as means ± SEM.

After i.p. inoculation of *Leptospira*, none of the challenged rats showed any clinical sign of disease during the whole experiment and all animals survived after three months. A non-parametric Kruskal-Wallis test was performed to compare the mean body weight percentage. The weight gain of challenged rats was not significantly distinct from that of the control rat group at the end of the experiment (from 37.93% to 41.70% of increase, H = 0.25, df = 3, p-value = 0.9, Fig. 1).

### Differential *Leptospira* urinary shedding from rats

*Leptospira* shedding in the urine of experimentally infected rats was addressed through culture and DNA detection on urine samples collected every week throughout the 3 months experiment. Overall, 286 urines samples excreted by 22 monitored rats could be analyzed. As shown in Table 1, no leptospires grew in culture medium inoculated with urine from any of the *L. mayottensis-* and *L. borgpetersenii-*infected rats. By contrast, live leptospires could be observed in culture medium inoculated with urine from 2 out of 7 *L. interrogans*-infected rats, starting from 4 weeks post-infection and until the end of the experiment. The shedding of *Leptospira* appeared intermittent as no cultivable leptospires could be detected at weeks 8 and 10 after infection.

**Table 1:** Rate of *Leptospira*-positive samples using either qPCR or cultures on urine of experimentally infected rats.

wpi	qPCR *Leptospira*-positive urine samples (%)			*Leptospira*-positive cultures (%)		
	*Li*	*Lm*	*Lb*	*Li*	*Lm*	*Lb*
1	1/7 (14.3)	0/8 (0.0)	2/7 (28.6)	0/7 (0.0)	0/8 (0.0)	0/7 (0.0)
2	3/7 (42.9)	6/8 (75.0)	4/7 (57.1)	0/7 (0.0)	0/8 (0.0)	0/7 (0.0)
3	5/7 (71.4)	1/8 (12.5)	4/7 (57.1)	0/7 (0.0)	0/8 (0.0)	0/7 (0.0)
4	2/7 (28.6)	0/8 (0.0)	1/7 (14.3)	1/7 (14.3)	0/8 (0.0)	0/7 (0.0)
5	2/7 (28.6)	1/8 (12.5)	0/7 (0.0)	2/7 (28.6)	0/8 (0.0)	0/7 (0.0)
6	1/7 (14.3)	1/8 (12.5)	0/7 (0.0)	2/7 (28.6)	0/8 (0.0)	0/7 (0.0)
7	2/7 (28.6)	1/8 (12.5)	0/7 (0.0)	2/7 (28.6)	0/8 (0.0)	0/7 (0.0)
8	1/7 (14.3)	1/8 (12.5)	0/7 (0.0)	0/7 (0.0)	0/8 (0.0)	0/7 (0.0)
9	1/7 (14.3)	0/8 (0.0)	1/7 (14.3)	2/7 (14.3)	0/8 (0.0)	0/7 (0.0)
10	2/7 (28.6)	0/8 (0.0)	0/7 (0.0)	0/7 (0.0)	0/8 (0.0)	0/7 (0.0)
11	2/7 (28.6)	0/8 (0.0)	0/7 (0.0)	2/7 (28.6)	0/8 (0.0)	0/7 (0.0)
12	1/7 (14.3)	1/8 (12.5)	0/7 (0.0)	2/7 (28.6)	0/8 (0.0)	0/7 (0.0)
13	1/7 (14.3)	0/8 (0.0)	0/7 (0.0)	1/7 (14.3)	0/8 (0.0)	0/7 (0.0)

wpi: week post-infection; *Li: L. interrogans; Lm: L. mayottensis; Lb: L. borgpetersenii*.

**Figure 2:**
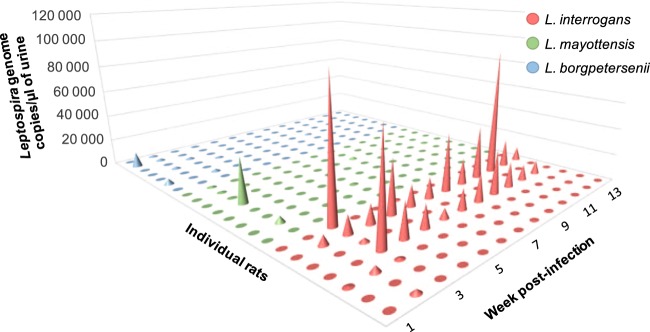
Urinary shedding of *Leptospira* from rats infected with either *L. interrogans* (red), *L. mayottensis* (green) or *L. borgpetersenii* (blue). Results are expressed for each individual rat as genome copies/µl of urine.

*Leptospira* DNA was detected in the urine of all *L. interrogans* infected rats, although discontinuously and with distinct concentration (Table 1, Fig. 2). Leptospira DNA was detected in urine samples during 10 and 12 consecutive weeks for 2 out of 7 *L. interrogans-*infected rats, with amounts ranging from 7,303.0 to 94,599.8 genome copies/µl. Urine samples of 1 additional rat tested positive during the first 3 weeks of the experiment. On the other hand, the detection of *Leptospira* DNA in the 4 remaining rats was sporadic and ranged from 230.2 to 100,746.5 genome copies/µl.

*Leptospira* DNA was detected less often and at lower levels in the urine of rats infected with *L. mayottensis* and *L. borgpetersenii* in comparison to individuals infected with *L. interrogans*. *Leptospira* DNA was sporadically detected in the urine of all *L. mayottensis*-infected rats with amounts ranging from 168.4 to 34,816.1 genome copies/µl.

Six out of seven *L. borgpetersenii*-infected rats shed *Leptospira* DNA in their urine. The detection was continuous in the first three weeks of the experiment for 2 rats and sporadic for the 4 remaining rats. Continuous DNA excretion ranged from 132.9 to 11,625.2 genome copies/µl while sporadic excretion ranged from 19.9 to 4,323.3 genome copies/µl.

Statistical analyses of the total number of copies detected for each rat over the study course revealed significant differences in the variability of *Leptospira* detection across strains (Bartlett’s test on Y_tot_: n = 22, K^2^ = 48.922, df = 2, *p* < 10^−10^). Hence, total individual *Leptospira* load varied across strains indicating significant variability across individual rats. A simple linear regression of the log-transformed total numbers revealed a weak effect of strain identity on the response (ANOVA: SS = 37.92, F_21,2_ = 2.673, p = 0.095).

The analysis based on linear mixed modeling of the (log-transformed) number of copies, Y, allowed us to account for significant zero-inflation in the data (229 cases without detection out of 286) as well as inter-individual variability. Four individual observations (out of 286), corresponding to zero values in the 2 rats showing high excretion of *L. interrogans* showed extreme values of response residuals. We present here the results after these observations were removed from analysis to improve the validity of the model, but we checked that the conclusions drawn from statistical inference did not depend on those observations. The final model retained was of the following form: Y ~ WPI + strain + (1 | strain / rat) for the conditional component, where WPI stands for week post-infection, strain indicates the strain and rat identity, and ~ (1 | strain / rat) for the zero-inflation component. Both random effects, in the conditional and in the zero-inflation components, improved the model, indicating significant variability across rats within strain groups with rats showing no or very weak excretion (Supplementary Table), as explained in the first part of the section. Regarding fixed effects, time after infection and strain identity showed both significant effects (ANOVA-II: WPI: Χ^2^ = 4.513, df = 1, p = 0.0336, strain: Χ^2^ = 8.705, df = 2, p = 0.013), negative for WPI (slope = −0.115, s.e. = 0.055) and with a significant positive coefficient for *L. interrogans* only (1.927, s.e. = 0.736, using contrasts by treatment with *L. borgpetersenii* as reference, *vs* 0.221, s.e. = 0.742 for *L. mayottensis*). No significant interaction was detected between WPI and strain.

### *Leptospira* spp. experimental infection does not necessarily induce chronic renal carriage and histological damage

**Figure 3:**
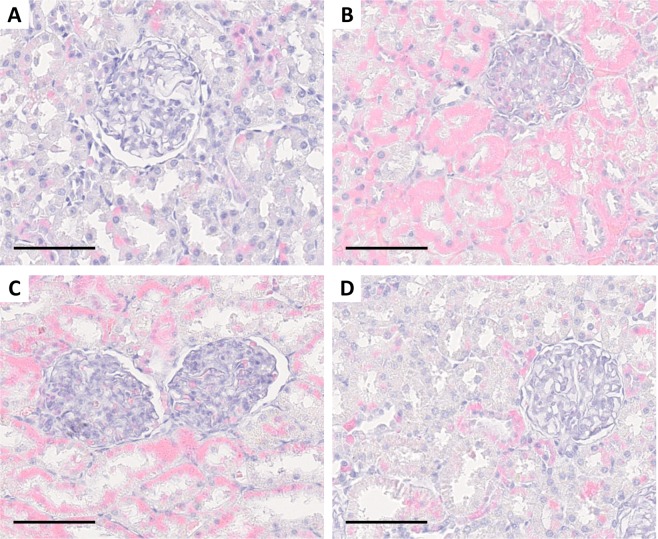
Kidney sections stained with hematoxylin-eosin from control rats **(A)** and rats infected with either *L. interrogans* **(B)**, *L. borgpetersenii* **(C)** or *L. mayottensis* **(D)**. Scale bars = 60 µm.

Following euthanasia, kidneys were screened by qPCR for *Leptospira* DNA. The test was negative for all rats infected with either *L. borgpetersenii* or *L. mayottensis*. Only those two rats chronically infected with *L. interrogans*, as shown by qPCR on urine, tested positive with loads of 108,670.8 ± 8178.1 and 201,132.3 ± 1077.7 copies/mg of kidney tissue. The kidneys from rats experimentally infected with all three *Leptospira* species were histologically studied using hematoxylin-eosin staining. Kidney sections showed no particular renal damage induced by the infection of rats with any of the three *Leptospira* isolates, including the two rats that were chronically shedding *L. interrogans*. Glomeruli and renal tubules of infected rats harbored the same phenotype as the control rats (Fig. 3).

## Discussion

In this study, we tested the ability of *Leptospira* isolates obtained from bats *(L. borgpetersenii)* and tenrecs *(L. mayottensis)*, two reservoirs endemic to SWIO islands, to replicate in a genetically different host via i.p. inoculation of these isolates in rats, a well-known *Leptospira* reservoir of *L. interrogans* worldwide including on several SWIO islands^4–6,15^.

As expected, our data indicate that rats do not develop any symptom of disease after inoculation of either of the three *Leptospira* species used in this study. However, they differed in their ability to develop an asymptomatic chronic renal carriage and *Leptospira* shedding in urine. This feature characterized only rats infected by the rat-borne *L. interrogans* cosmopolitan isolate but none of the rats infected by the tenrec-borne *L. mayottensis* and the bat-borne *L. borgpetersenii* isolates. Indeed, *Leptospira* DNA was detected during 10 to 12 consecutive weeks in the urine of two out of seven *L. interrogans*-infected rats while more sporadically and for not more than 3 consecutive weeks in rats infected with either *L*. mayottensis or *L. borgpetersenii*. Moreover, live leptospires could grow in cultures of urine samples obtained from the two aforementioned *L. interrogans*-infected rats during several consecutive weeks, whereas urine samples obtained from *L. mayottensis*- and *L. borgpetersenii*-infected rats did not lead to any detectable growth of *Leptospira*. When controlling for inter-individual variability, which includes the absence of shedding in some rats, the injected *Leptospira* isolate was retained in the final linear mixed model. In addition, strain identity shows a significant effect when analyzing fixed effect, with a significant positive coefficient found for *L. interrogans* only. Of note, the two rats harboring chronic infection with *L. interrogans* were housed in the same cage with 2 other rats seemingly not chronically infected with *Leptospira*. The two chronically infected rats shed bacteria with comparable kinetics throughout the experiment. Hence direct or indirect horizontal contamination was likely not involved in renal colonization in our experiment.

No particular lesion was observed in the kidneys of any rat following infection, which is not unexpected as rats are resistant to *Leptospira* infection^30,31^. Indeed, previous studies have already reported the absence of renal lesions in rats experimentally infected by *L. interrogans*^30,32^, although such rats can also harbor nephritis and cellular infiltrates^23,24^.

One should note that in our study, the rate of renal colonization induced in rats by *L. interrogans* isolate was lower (2 out 7 rats) than previously reported in comparable experiments also carried out using Wistar rats^22,24,33^. In addition, the kinetics of shedding (4–5 weeks for detection of live bacteria) was slower than reported in these previous studies (only one week despite lower bacterial inoculums) (Athanazio *et al*., 2008). These results might reflect a lower virulence of the *L. interrogans* used in our study. Similarly, one may consider that the inability of *L. borgpetersenii* and *L mayottensis* recovered from endemic hosts to colonize rats’ kidneys may also reflect an attenuated virulence of these isolates. In a previous study^27^, we have shown that the *L. interrogans* isolate, the same than the one used in the present study, was (i) significantly more virulent than *L. mayottensis* and *L. borgpetersenii* isolates and (ii) able to colonize kidneys after experimental infection of the highly sensitive hamsters more efficiently than did the *Leptospira* isolates recovered from endemic hosts.

An alternative explanation of our observations is that the absence of renal colonization in rats by the endemic strains could result from some natural incompatibility possibly genetically determined between the bacterium and this alternative vertebrate host: the *L. mayottensis* and *L. borgpetersenii* isolates may not be able to adhere to cells of the host kidney tubules of rats and hamsters and organize bacterial biofilms^27,34^. Noteworthily, *L. mayottensis* has been recently detected in rats from Madagascar but mostly as co-infections with *L. interrogans*^19^. Hence, a primary infection in rats with *L. interrogans* may be a prerequisite for a secondary infection with *L. mayottensis*. In other words, *L. mayottensis* may be able to form a mixed biofilm with *L. interrogans*, but unable to form a mono species biofilm. This could be tested experimentally by inoculating *L. mayottensis* into rats that are already chronically shedding *L. interrogans*, or by injecting a mixture of *L. interrogans* and *L. mayottensis*.

Studies on Friend virus virulence evolution have shown that viral fitness could be impaired by several passages of the virus through hosts of different genotypes. By contrast, several passages of the pathogen through hosts of identical genotypes led to an increase in pathogen fitness^35^. Thus, the *Leptospira* isolates obtained from bat and tenrec specimens may have a decreased fitness in rats, hence impairing chronic infection in this species. Further investigations are needed to test whether serial passages of a specific *Leptospira* lineage through a novel animal target (such as rats) can allow its adaptation to this alternative host.

In conclusion, *Leptospira* isolates obtained from small volant and terrestrial mammals endemic to western Indian Ocean were not experimentally able to colonize rats’ kidneys, in keeping with the host specificity observed in natural conditions^8,15^. Our results suggest that the strong host specificity of *Leptospira* species/lineages endemic to SWIO islands towards their mammalian hosts^8^ is at least in part related to host genetics. These data certainly do not rule out the existence of ecological factors. Indeed, frequent inter-species physical contacts such as those reported between two bat species, namely *Myotis goudoti* and *Miniopterus* spp., have been proposed to facilitate *Leptospira* host switches^13,36^. However, presented data support the existence of genetic determinants involved in host-*Leptospira* specificity. Symmetrical experiments, *i.e*. challenging bats and tenrecs with all three isolates, would bring further relevant information although such experiments require accessing to lab colonies of bats and tenrecs, which are very few worldwide. Other biological characteristics of the *Leptospira* isolates may also account for our results such as attenuated virulence;^27^ low growth rate of *Leptospira* may also hamper *L. mayottensis* and *L. borgpetersenii* from chronically infecting mammalian host other than their natural reservoir. Further experiments are needed to solve this issue and unravel factors that may be at play in such interactions.

## Supplementary information

Supplementary Table
