# Supplementary material for: Pathogenic Leptospira and their animal reservoirs: testing host specificity through experimental infection
Source: Sci Rep. 2020 Apr 29;10:7239. doi: 10.1038/s41598-020-64172-4 (PMC7190861; doi:10.1038/s41598-020-64172-4)
Supplement: Supplementary file 1 — Supplementary Table [file 41598_2020_64172_MOESM1_ESM.docx]

**Pathogenic *Leptospira* and their animal reservoirs: testing host specificity through experimental infection**

**Colette Cordonin, Magali Turpin, Matthieu Bringart, Jean-Loup Bascands, Olivier Flores, Koussay Dellagi, Patrick Mavingui, Marjolaine Roche, Pablo Tortosa**

**Supplementary Table.** Variance estimates associated with the two components of the linear mixed model calibrated on the (log-transformed) number of copies of Leptospira strains in urine samples (n = 282 samples on 22 rats). Although the strain contribution was very low in the conditional part of the model, it was retained because of the nature of rats identity nested, and not crossed, within strains.

|  | Conditional | Zero-inflation |
| --- | --- | --- |
| Strain (Cond.) | 3.440.10^-9^ | 0.2635 |
| Rat : Strain | 1.063 | 1.5591 |
| Residuals | 1.3516 | |
